# Self-control for maintaining healthy lifestyle behaviors associated with serum lipid levels in preadolescent children in Japan: a cross-sectional study

**DOI:** 10.3389/fpubh.2025.1678686

**Published:** 2025-12-04

**Authors:** Kotomi Yamashita, Hiromi Kawasaki, Satoko Yamasaki, Sae Nakaoka, Bethel Fekede Menuta

**Affiliations:** Department of Nursing, Graduate School of Biomedical and Health Sciences, Hiroshima University, Hiroshima, Japan

**Keywords:** serum lipid, lifestyle behavior, health behavior, pre-adolescence, children, self-control

## Abstract

**Introduction:**

Serum lipid levels influence long-term health outcomes in children and self-control is crucial for healthy behavioral habits. This study focused on preadolescent children aged 9–10 years, who are developing self-control. Moreover, the study aimed to examine the relationship between serum lipid levels and self-control of lifestyle behavior to identify the behaviors that warrant prioritization for self-control.

**Methods:**

The study included 545 children. Non-high-density lipoprotein cholesterol (non-HDL-C) is an indicator of current and future health status in children. Differences in non-HDL-C values were tested based on the self-control status of healthy behaviors. Given that behavioral performance is interrelated, logistic regression analysis was performed.

**Results:**

Non-HDL-C levels were lower in groups that frequently consumed fried food and in those with poor physical condition and mood on Mondays. In logistic regression, lower serum lipid levels were associated with sufficient exercise time, poor physical condition, and mood on Mondays, lower body weight, and being male. Avoiding snacking while watching television may also be associated with lower non-HDL-C levels.

**Discussion:**

Self-control of sufficient exercise time and adherence to snacking rules are pivotal for self-control among preadolescent children, and enhancing self-control in these behaviors can be critical in educational and intervention strategies aimed at improving health outcomes.

## 1 Introduction

Serum lipid levels during childhood tend to persist through adolescence and adulthood (1, 2), and consistent high serum lipid levels increase the risk of developing atherosclerotic disease later in life. Adopting healthy lifestyle behaviors early in childhood is important because high serum lipid levels among children have long-term health implications (2, 3). According to the social cognitive theory, individual behavior is shaped by the reciprocal interaction of “personal cognitive influence on behavior,” “social and environmental influence on behavior,” and “supporting behavior factors” (4). Parents and schoolteachers must mitigate and influence children’s behaviors and environments to facilitate the adoption of healthy behaviors during their growth and development (i.e., “social and environmental influence on behavior”). However, continuously overseeing all aspects of a child’s life is not advisable, as children need to develop the capacity to autonomously control their lifestyle behaviors.

In prior studies, the term “self-control” has been used interchangeably with self-regulation (5, 6). In this study, we have used the term self-control. Higher self-control skills are associated with healthy lifestyle behavior performance, such as reduced screen time (7), even in the absence of parental oversight. In these studies, self-control was evaluated through the management of emotions, impulsivity, and delayed gratification tasks. Research indicates that self-control in dietary behavior encompasses the suppression of the inclination to select unhealthy foods in favor of healthier alternatives (6, 8, 9), postponement or avoidance of the immediate impulse to consume food, and restraint from eating beyond energy requirements (9). Given the interconnected nature of lifestyle behaviors, a comprehensive examination is required. Nonetheless, to the best of our knowledge, no study has investigated self-control in the context of multiple lifestyle behaviors.

The preadolescent stage is a transitional period during which children begin to develop autonomy in their lifestyle behaviors. As children grow and develop, management gradually shifts from the parents to the children themselves (5). However, in the latter half of puberty, children’s resistance to adult authority can make it difficult for parents to manage their children’s daily lifestyle behaviors. In addition, “unhealthy” behaviors, such as prolonged sitting or screen time, insufficient physical activity, and the consumption of high-fat foods, which are less influenced by parental oversight, often begin and persist thereafter (10). Therefore, enhancing awareness among children and promoting self-control in health behaviors and recognizing at ages 9–10 during preadolescence are important for sustained control of lifestyle behaviors.

The specific behaviors that preadolescent children should control and the extent of control necessary for optimal health behavior performance remain unclear. Identifying which behaviors among the various lifestyle behaviors warrant particular control will facilitate more effective education and intervention strategies. The current study is the first to comprehensively examine the control of serum lipid levels, a significant determinant of current and future health outcomes, in conjunction with healthy lifestyle behaviors. Furthermore, the study extracted lifestyle behavior variables crucial for current and future health outcomes and inferred the level of self-control over these behaviors utilizing children’s responses to a lifestyle survey. Modifiable behaviors and recommended standards for children’s health behaviors were duly considered during the selection of lifestyle behavior variables (11, 12). Furthermore, acknowledging that children’s self-control over daily behaviors is influenced by parents and teachers, items incorporating social and environmental factors in line with social cognitive theory (4) were also extracted.

This study aimed to comprehensively examine the relationship between low serum lipid levels and healthy lifestyle behavior control in preadolescent children. In addition, the study aimed to specify items that should be prioritized in education and intervention, particularly for children transitioning to self-control of their lifestyle behaviors, by identifying the lifestyle behaviors that should be particular control.

## 2 Methods

### 2.1 Study design

This quantitative, cross-sectional study analyzed secondary data from a health promotion program conducted in City B, Hiroshima Prefecture, Japan.

### 2.2 Study sample

This study is a secondary analysis of data collected from 2014 to 2016 as part of a health promotion program conducted by the local government and included serum lipid levels, physical measurements, and lifestyle survey responses from fourth-grade children (aged 9–10 years). The program targeted all students in the fourth grade children attending public elementary schools in the city and aimed to promote healthy lifestyle habit development in children during a critical period of habit formation, to prevent the onset of lifestyle-related diseases in adulthood. A questionnaire survey was administered to assess daily lifestyle habits and evaluate the health education provided. Blood tests were conducted to objectively assess the children’s health status. Data were obtained from 227 children in 2014, 243 in 2015, and 232 in 2016, for a total of 702 children. All data were combined and analyzed as a single dataset because no significant differences were observed between the years.

#### 2.2.1 Inclusion and exclusion criteria for analysis

**Figure 1:**
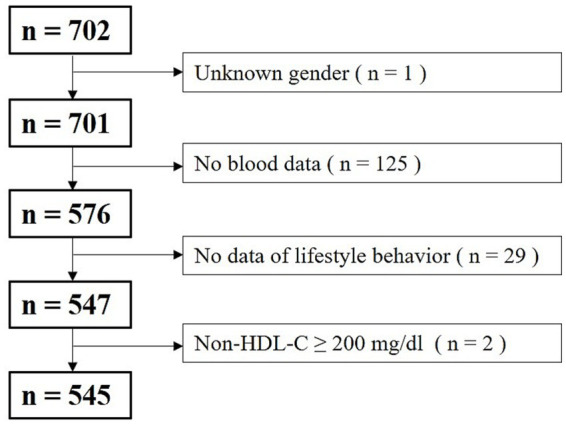
Flowchart depicting the inclusion of study sample.

The inclusion criteria for the subjects analyzed in this study were lifestyle survey responses and blood data. The following exclusions were applied: participants of unknown sex (*n* = 1), missing blood data (*n* = 125), incomplete responses to questions regarding daily lifestyle management (*n* = 29), and extremely high non–high-density lipoprotein cholesterol (non-HDL-C) levels (≥200 mg/dL, *n* = 2). After exclusion, the final sample size was 545 participants (77.6%). A flowchart outlining participant inclusion is shown in Figure 1.

#### 2.2.2 Sample size evaluation

Regarding the rationale for the sample size, the minimum effective sample size was calculated based on the number of variables in the final model of logistic regression analysis. The number of events per variable was considered to be at least 20 (13). In this study, 13 variables were finally included in the logistic regression model; the minimum effective sample size was *n* = 13 × 20 = 260 cases. Therefore, *n* = 270 for less than the 50th percentile of non-HDL-C and *n* = 275 for more than the median, both exceeding *n* = 260, thus meeting the sample size requirement. These data were deemed usable because they can withstand analysis.

#### 2.2.3 Target sample

The study included fourth-grade students aged 9–10 years. This age range represents a critical transitional period during which children begin to independently control healthy lifestyle behaviors, making it an ideal group for examining the relationship between self-control of healthy lifestyle behaviors and serum lipid levels. Although serum lipid levels generally increase with age, they temporarily decrease during puberty (14–16). Therefore, serum lipid levels at ages 9–10 years may more accurately reflect the influence of children’s daily lifestyle behaviors.

### 2.3 Self-control of healthy lifestyle behaviors

This study used responses from a lifestyle survey completed by the children to assess children’s self-control of healthy lifestyle behaviors. The lifestyle survey was designed to assess health education initiatives implemented in elementary schools and to reflect lifestyle habits. The original questionnaire consisted of 57 items. The response scale was set according to the question, either as a four-point scale (e.g., “very much,” “somewhat,” “not much,” “not at all”) or a two-point scale (yes/no). The researchers originally drafted the questionnaire, which was reviewed by local public health nurses, the Board of Education, principals, and school nurses. Elementary school teachers confirmed that the content was understandable to the children to ensure its suitability for the target age group. Then, children who were not part of the main survey were asked to answer the questionnaire on a trial basis, and the wording was adjusted as required. The questionnaire contents were finalized by the local government’s health promotion program; therefore, internal consistency was not assessed. However, before analysis, the Cronbach’s alpha for the survey items in Tables 1, 2 used in this study was 0.66. The questionnaires were distributed and collected by homeroom teachers.

**Table 1:** Non-HDL-C levels and self-control of healthy lifestyle behaviors.

Self-control of lifestyle behaviors	Non-HDL-C < 111 mg/dL		Non-HDL-C ≥ 111 mg/dL	
	*n*	(%)	*n*	(%)
Screen time (hours per day)				
<2 h	172	49.4	176	50.6
**≥**2 h	103	52.3	94	47.7
Sleep time (hours per day)				
<9 h	164	52.6	148	47.4
**≥**9 h	111	47.6	122	52.4
Exercise				
Exercise time (hours per day)				
≥1 h	180	51.0	173	49.0
<1 h	95	49.5	97	50.5
Having time for exercise				
Sufficient time	132	46.8	150	53.2
Not so much	143	54.4	120	45.6
Do you want to exercise more?				
Yes	168	51.1	161	48.9
No	107	49.5	109	50.5
Physical condition and mood on Mondays				
Very good	130	54.9	107	45.1
Not good/poor	145	47.1	163	52.9

Non-HDL-C, non–high-density lipoprotein cholesterol.

**Table 2:** Non-HDL-C levels and self-control of eating behaviors.

Self-control of eating behaviors	Non-HDL-C < 111 mg/dL		Non-HDL-C ≥ 111 mg/dL	
	n	(%)	n	(%)
Breakfast				
Every day	217	47.8	237	52.2
Missed meals	58	63.7	33	36.3
Second helping of school lunch				
Rarely	114	50.9	110	49.1
Sometimes	161	50.2	160	49.8
Frequency of fried food consumption				
Not much	96	54.2	81	45.8
Frequently or sometimes	179	48.6	189	51.4
Snacks after dinner				
Rarely	153	52.0	141	48.0
Sometimes eat	122	48.6	129	51.4
Being mindful not snacking while watching television				
Always	127	47.9	138	52.1
Sometimes or never	148	52.9	132	47.1
Thinking about food habits for health				
Always	61	51.7	57	48.3
Sometimes or never	214	50.1	213	49.9
Taking care to chew meals				
Always	81	52.3	74	47.7
Sometimes or never	194	49.7	196	50.3
Being mindful of eating various foods				
Always	94	51.6	88	48.4
Sometimes or never	181	49.9	182	50.1
Avoiding too much juice				
Always	97	49.7	98	50.3
Sometimes or never	178	50.9	172	49.1
Avoiding too much sweets				
Always	127	49.8	128	50.2
Sometimes or never	148	51.0	142	49.0
Avoiding highly salty foods				
Always	144	53.9	123	46.1
Sometimes or never	131	47.1	147	52.9
Not being picky about food				
Always	122	50.8	118	49.2
Sometimes or never	153	50.2	152	49.8
Being mindful of eating vegetables				
Always	137	50.7	133	49.3
Sometimes or never	138	50.2	137	49.8

Non-HDL-C, non–high-density lipoprotein cholesterol.

Based on the survey results, items related to self-control of healthy lifestyle behaviors that were considered relevant for children’s current and future health were identified. During the extraction, items indicated in the guidelines for healthy lifestyle behaviors in children (11, 12), as well as factors considered as social/environmental influences on behavior according to the social cognitive theory (4) were included. Tables 1, 2 present the extracted lifestyle variables, including physical activity (exercise time hours/per day, having sufficient exercise time, and wanting more exercise), eating behaviors (frequency of fried food consumption, eating vegetables, avoid being picky, chewing thoroughly, and thinking about meals for health), rules for snacking (avoid eating snacks while watching television, avoid snacks after dinner, and avoid too much juice), screen time, sleep time, and Monday’s physical condition or mood. These variables are important for the current and future health outcomes of the child. The variable “physical condition and mood on Mondays” was included because it was considered to reflect children’s self-control status over the weekend behavior.

### 2.4 Physical measurements

Elementary school teachers measured the height and weight of the children using standardized scales at each school and recorded the lifestyle questionnaire. In accordance with the School Health and Safety Act Enforcement Regulations in Japan, children’s height and weight are measured and recorded annually, and these recorded values were used in this study.

### 2.5 Blood data

Blood samples were collected via venipuncture at each elementary school. Blood tests were conducted by a contractor commissioned by the local government. All blood test costs were fully covered by the local government. In Japan, children are encouraged from early childhood to eat breakfast daily. Given the educational emphasis on this habit, children and their parents were instructed to eat breakfast as usual on the day of the blood test. Blood samples were collected at approximately 11:00 h, before lunch, to minimize the impact of breakfast on the test results.

#### 2.5.1 Non-HDL-C as a serum lipid levels assessment

In this study, serum lipid data, which are important for assessing the risk of atherosclerotic diseases, were analyzed using the collected blood samples. Non-HDL-C is the value obtained by subtracting high density lipoprotein cholesterol levels from total cholesterol levels. Non-HDL-C contains all remnants that cause arteriosclerosis, making it superior for assessing the risk of arteriosclerosis. Low non-HDL-C levels reduce the risk of arteriosclerosis and improve health outcomes.

In addition, non-HDL-C levels are less affected by diet than low-density lipoprotein cholesterol levels (17, 18). The use of non-HDL-C, which is relatively unaffected by food intake, is recommended, because blood sampling during childhood, a period of growth and development, is difficult to conduct under fasting conditions (2, 17, 19, 20). Therefore, non-HDL-C was evaluated in this study.

### 2.6 Analysis method

#### 2.6.1 Physical measurements

The body mass index was calculated using height and weight values. The participants’ height and weight data were aggregated by sex, considering sex differences in growth and development.

#### 2.6.2 Relationship between non-HDL-C levels and self-control of healthy lifestyle behavior

The hypothesis of this study was: “Self-control of healthy lifestyle behaviors among preadolescent children is associated with maintaining low serum lipid levels.” The following analyses were performed to test this hypothesis.

#### 2.6.3 Testing difference in non-HDL-C levels based on self-control of healthy lifestyle status

The differences in serum lipid levels between the two groups classified by daily lifestyle management status were examined. The two groups for self-control status of healthy lifestyle behaviors were divided so that each group contained half of the participants. Screen time and duration of sleep were categorized according to the children’s health behavior guidelines (11, 12). Normality was tested using the Shapiro–Wilk test and histograms of non-HDL-C. If the distribution of non-HDL-C in each of the two groups was assumed to be normal, then an independent *t*-test was used; if normality was rejected, the Wilcoxon rank-sum test (Mann–Whitney U test) was applied. For the independent *t*-test, mean ± standard deviation, 95% confidence interval (95% CI), and *p*-value of non-HDL-C in each group were calculated. For the Wilcoxon rank-sum test, the median and interquartile range (IQR) and the effect size and *p*-value were calculated for each group.

#### 2.6.4 Logistic regression analysis

As lifestyle behaviors are interrelated, comprehensive assessment is necessary. Therefore, logistic regression analysis was chosen. This study targeted “healthy” children living in the community, and as most of these children had “standard” serum lipid levels and their self-control status of lifestyle behaviors was being evaluated, logistic regression analysis was used instead of multiple regression analysis, with which the “values” of serum lipids would be predicted. The dependent variable for logistic regression analysis was set as non-HDL-C values (below median = 1, median or above = 0.) Independent variable included the self-control status of healthy lifestyle behaviors. In this study, if all variables related to daily lifestyle management were converted into dummy variables for each of the four response options, the number of independent variables would have been too large to perform logistic regression analysis (13). Therefore, the self-control status of healthy lifestyle behaviors was converted into two categories based on the distribution of responses. Moreover, serum lipid levels tend to increase with weight gain, because there are sex differences in these levels; hence, sex (male = 1, female = 0) and weight (below median = 1, median or above = 0) were also included in the analysis. Odds ratios (OR) and 95% CI were calculated.

##### 2.6.4.1 Multicollinearity

In selecting independent variables, if the correlation coefficient between two independent variables was *r* > 0.8, the content of the self-control of lifestyle behaviors was similar, or the variance inflation factor (VIF) exceeded five, then multicollinearity was considered a potential issue (21). In such cases, one of the correlated variables was excluded from the analysis. The variables “avoiding too much snacks” and “avoiding too much juice” were significantly correlated (*r* = 0.515, *p* < 0.001) and were judged to have similar content in terms of self-control of lifestyle behavior. Therefore, “avoiding too much snacks” was removed from the set of independent variables. All VIFs for the maintained variables were checked, and the VIF values were within the range of 1–2, indicating no concerns regarding multicollinearity based on VIF.

##### 2.6.4.2 Determination of independent variables

Logistic regression analysis was conducted using a variable reduction method based on likelihood ratio tests. The optimal model was selected through discussion among the authors, accounting for model fit indicators (positive predictive rate, Hosmer-Lemeshow test, and Nagelkerke R^2^). In addition, in the selected model, variables previously reported as key lifestyle behaviors associated with serum lipids, such as screen time, sleep duration, physical activity, dietary management, body weight, and sex, were confirmed to be included as independent variables (2, 5, 6, 11, 12, 22). During the analysis, if a variable was removed through the reduction process, then such a variable was manually re-entered as an independent variable. All analyses were performed using IBM SPSS Statistics version 25. The significance level was set at *p* < 0.05.

### 2.7 Ethical considerations

This study was conducted in accordance with the Declaration of Helsinki and was approved approval from the Ethical Committee for Epidemiology of Hiroshima University (protocol code: E-535-2). All data were anonymized, with physical measurements, blood test results, and lifestyle survey response linked through identification numbers to prevent the identification of individuals. This study is a based on the secondary use of data derived from a health promotion program. Before implementing the health promotion program, local government officials written and oral briefings to children and their parents. For blood testing, a video demonstrating the blood collection procedure was also provided to help prepare the children. Participation in the blood tests was voluntary, and it was clearly explained that those non-participation would entail no disadvantages. Both parental and child consent were required for participation in the blood test. Furthermore, participants were informed that all data would be anonymized, detailed analysis would be conducted at the authors’ affiliated institutions, and that personal information would be protected. Consent for the secondary use of data obtained from the health promotion program was obtained from local government officials.

## 3 Results

### 3.1 Basic information

The median for non-HDL-C in the study population was 111 mg/dL. Among the study subjects, 270 had non-HDL-C levels below the median, whereas 275 had levels at or above the median.

The self-control status of lifestyle behaviors of the two groups (non-HDL-C below vs. at or above the median) is presented in Table 1. The self-control status of eating behaviors is presented in Table 2. The physical measurements and serum lipid levels of the subjects are presented in Table 3.

**Table 3:** Physical measurements and serum lipid levels of study subjects.

Physical measurements and serum lipids	Male (*n* = 262)		Female (*n* = 283)	
	Mean ± SD	Median	Mean ± SD	Median
Somatometry				
Height (cm)	135.53 ± 6.01	135.60	135.75 ± 6.68	135.50
Weight (kg)	32.63 ± 6.68	31.20	31.72 ± 6.63	30.60
Body mass index (kg/m^2^)	17.6 ± 2.79	16.90	17.11 ± 2.63	16.67
Serum lipid levels				
Total cholesterol (mg/dL)	172.57 ± 24.80	170.50	175.41 ± 24.63	175.00
Triglycerides (mg/dL)	79.24 ± 46.72	70.00	78.69 ± 38.89	70.00
HDL cholesterol (mg/dL)	61.29 ± 10.75	61.00	61.32 ± 12.16	60.00
LDL cholesterol (mg/dL)	94.84 ± 22.50	91.50	97.58 ± 23.07	96.00
*Non-HDL cholesterol (mg/dL)	111.27 ± 23.57	109.00	114.09 ± 23.25	113.00

*Non-HDL cholesterol = Total cholesterol − HDL cholesterol (mg/dL). SD, standard deviation; HDL, high-density lipoprotein; LDL, low-density lipoprotein.

### 3.2 Differences in non-HDL-C levels based on self-control status of lifestyle behaviors

**Figure 2:**
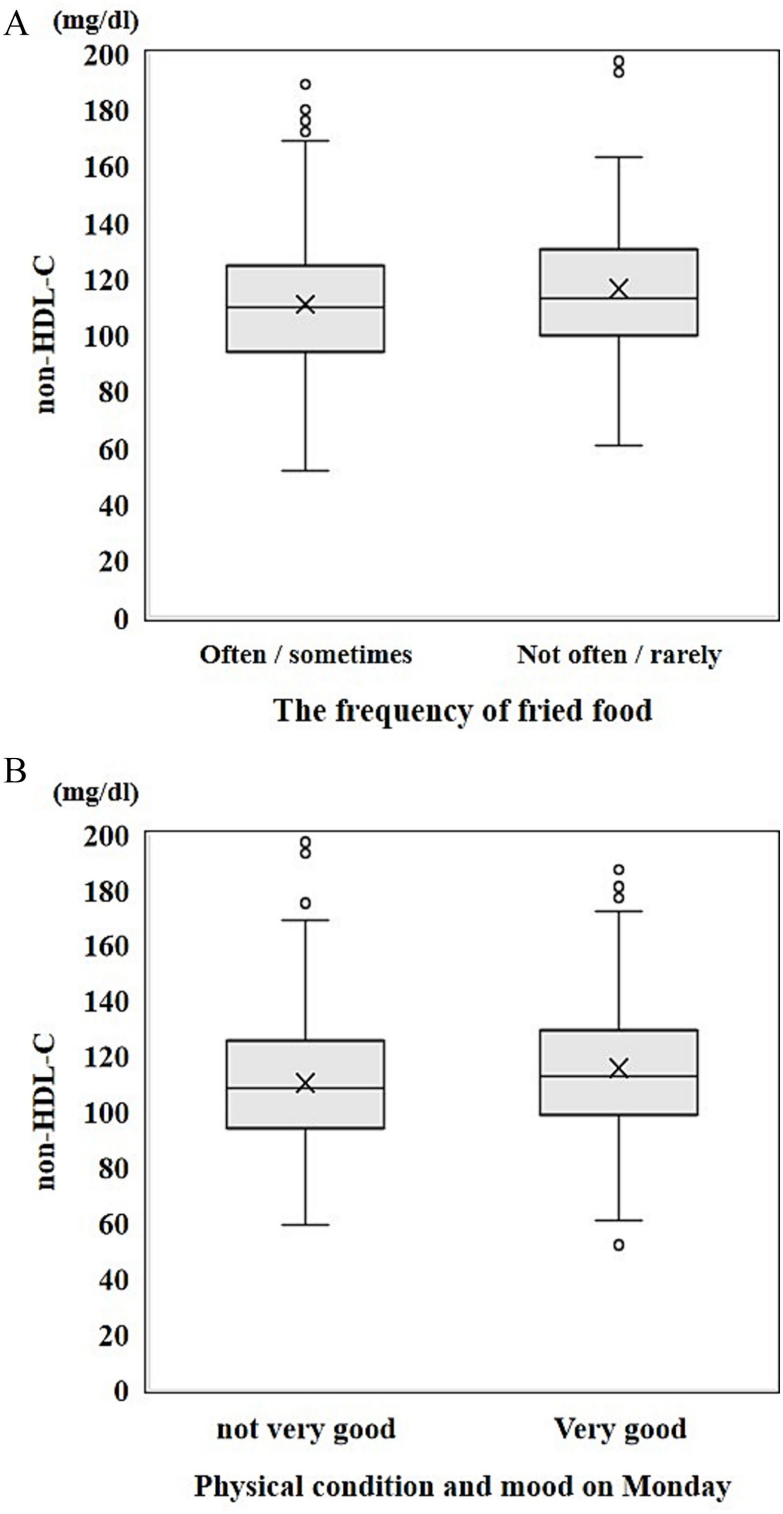
Difference in non-HDL-C levels between the two groups based on **(A)** frequency of fried food consumption and **(B)** physical condition and mood on Mondays.

Non-HDL-C levels were compared between two groups based on the self-control status of lifestyle behaviors. The Shapiro–Wilk test indicated that data were not normally distributed; therefore, the Wilcoxon rank-sum test was used. The group that reported “often” or “sometimes” eating fried foods (median = 110 mg/dL, IQR = 94–124 mg/dL) had significant lower non-HDL-C levels compared with those who reported “not often” or “rarely” consuming fried foods (median = 113 mg/dL, IQR = 100–130 mg/dL; *p* = 0.008, *r* = 0.11) (Figure 2A) and in those who reported “not good” or “poor” physical condition or mood on Mondays (median = 108, IQR = 94–125) compared with those who reported “very good” (median = 113, IQR = 99–129; *p* = 0.010, *r* = 0.11) (Figure 2B).

### 3.3 Relationship between non-HDL-C levels and self-control status of lifestyle behavior by logistic regression analysis

Table 4 shows the results of logistic regression analysis examining the relationship between non-HDL-C levels and the self-control status of lifestyle behaviors. The Hosmer–Lemeshow test indicated a good model fit (*p* = 0.171), positive predictive rate was 59.8%, and Nagelkerke R2 was 0.068.

**Table 4:** The serum lipid levels and the self-control of healthy lifestyle behavior status in logistic regression analysis.

Variable	B	OR	95% CI	*p*
Screen time (1: ≤ 2 h per day)	0.095	1.10	0.757–1.599	0.617
Exercise time (1: ≥1 h per day)	−0.122	0.885	0.606–1.293	0.528
Having time for exercise (1: enough time)	0.423	1.527	1.049–2.223	0.027*
Want to exercise more (1: yes)	−0.193	0.825	0.560–1.207	0.322
Being mindful not to snacking while watching television (1: always mindful)	0.373	1.452	0.991–2.127	0.055
Snacking after dinner (1: rarely)	−0.116	0.890	0.615–1.289	0.539
Avoiding too much juice (1: always careful)	0.128	1.136	0.764–1.691	0.528
Avoiding highly salty foods (1: always careful)	−0.266	0.766	0.526–1.115	0.164
Frequency of fried food consumption (1: not much/rarely)	−0.261	0.771	0.528–1.124	0.176
Sleep duration (1: ≥9 h per day)	−0.193	0.825	0.580–1.173	0.284
Physical condition and mood on Mondays (1: very good)	−0.423	0.655	0.454–0.947	0.024*
Weight (1: below the 50th percentile)	0.451	1.570	1.103–2.235	0.012*
Sex (1: male)	0.371	1.449	1.007–2.086	0.046*

Dependent variable: non-HDL cholesterol level (1 = below the median). *p < 0.05. HDL, high-density lipoprotein; OR, odds ratio; CI, confidence interval.

Non-HDL-C levels below the median were significantly associated with the following lifestyle management factors: “having sufficient exercise time” [*B* = 0.423, OR: 1.527, 95% confidence interval (CI): 1.049–2.223, *p* = 0.027], “physical condition and mood on Mondays are very good” (*B* = − 0.423, OR: 0.655, 95% CI: 0.454–0.947, *p* = 0.024), “body weight below the median” (*B* = 0.451, OR: 1.570, 95% CI: 1.103–2.235, *p* = 0.012), and “being male” (*B* = 0.371, OR: 1.449, 95% CI: 1.007–2.086, *p* = 0.046), and “mindful not to snack while watching TV” (*B* = 0.373, OR: 1.452, 95% CI: 0.991–2.127, *p* = 0.055) may also be associated.

## 4 Discussion

### 4.1 Main findings

In this study, participants who frequently consumed fried foods (compared with those who rarely ate them) and those who reported poor physical condition or mood on Mondays (compared with those who reported very good condition) had significantly lower non-HDL-C levels than those in the comparison groups. In addition, logistic regression analysis revealed the following factors as being associated with non-HDL-C levels below the median of the study population: “having sufficient exercise time,” “poor physical or mental condition on Mondays,” “body weight below median” and “being male,” and “mindful not to snack while watching television (adherence to the rules for snacking)” may also be associated.

### 4.2 Interpretation

#### 4.2.1 Physical measurements

In a previous study, non-HDL-C levels in Japanese 9-year-olds were as follows: male (*n* = 26,654) had a median of 104 mg/dL, whereas female (*n* = 51,503) had a median of 109 mg/dL (18). In the present study population, the median non-HDL-C levels were 109 mg/dL for males and 113 mg/dL for females, which are roughly the same values reported in broader studies of Japanese children.

#### 4.2.2 Differences in non-HDL-C levels based on self-control status of lifestyle behaviors

Non-HDL-C levels were significantly lower in children who frequently consumed fried foods compared to those who rarely consumed such foods. Furthermore, children who reported feeling in poor physical condition or mood on Mondays exhibited lower non-HDL-C levels than those who reported feeling very well. We have discussed the key lifestyle factors that 9- to 10-year-old children should manage for maintaining optimal health.

##### 4.2.2.1 Fried food consumption

Non-HDL-C levels were significantly lower in children who consumed fried foods often or sometimes compared with those who rarely ate them. This finding contrasts with those of a previous study, which showed that consuming fried foods four or more times per week is associated with an increased risk of lifestyle-related diseases (23, 24). The relatively low effect size observed in this study may be attributed to the reliance on self-reported data from children, which raises the possibility of recall bias, as 9- to 10-year-olds may not accurately remember their dietary intake (9). Previous studies highlighted the need to consider the complexities of fried food consumption. For instance, meals using polyunsaturated fats, such as olive oil, are considered beneficial for health (23, 25). The control of eating behavior is subject to a multitude of influences, including an individual’s physiological constitution, dietary restrictions, eating patterns and quantities consumed by peers (normative influence), emotional states and stress levels, distracting stimuli, caloric deficits, and food insecurity (hunger) (9). This study did not account for these confounding factors; thus, it cannot definitively determine whether the frequency of fried food consumption should be controlled or adjusted for individuals with low serum lipid levels. Future research should incorporate an analysis of parental responses and the associated factors influencing the consumption of fried foods.

##### 4.2.2.2 Physical condition and mood on Mondays

Children who reported poor physical condition and mood on Mondays had significantly lower non-HDL-C levels. Their physical condition and mood on Monday reflected the self-control status of lifestyle behavior over the weekend, which contradicts our hypothesis that those who feel well on Mondays would have lower serum lipid levels. The effect size observed was minimal, and the analysis indicated the potential influence of confounding variables in the relationship between Monday’s physical condition or mood and serum lipid values. Compared with other days of the week, people are more likely to feel unwell on Mondays (26). Although high non-HDL-C levels including remnant cholesterol, may increase the long-term risk of atherosclerotic disease, from a short-term perspective, they may contribute to improved subjective mood and physical condition. Based on the results of this study, drawing conclusions about the relationship between self-control of weekend healthy lifestyle behavior and serum lipids is not possible. Further analysis incorporating additional data on weekend behaviors is required.

#### 4.2.3 Comprehensive relationship between non-HDL-C levels and self-control of healthy lifestyle behaviors

Logistic regression analysis showed that lower non-HDL-C levels are associated with “having sufficient exercise time,” “poor physical condition and mood on Mondays,” and “Being mindful not to eat snacks while watching television (adhering to rules for snacking)” may be related.

##### 4.2.3.1 Having sufficient exercise time

Sufficient exercise time was associated with lower non-HDL-C levels. Longer exercise duration is associated with lower serum lipid levels and improved health outcomes in children (3, 27, 28). Previous studies have highlighted the importance of exercise in children’s health outcomes (3, 27, 29). This study demonstrates that effective control and adaptation to allocate time for exercise is correlated with reduced serum lipid levels. For the self-control of having sufficient time for exercise, providing knowledge to children on the relationship between health and exercise, adjusting the environment and schedule to make it possible or easier to exercise by parents (4, 5) and supporting children’s efforts to reduce sedentary time and engage in physical activity are important (30). Furthermore, children aged 9–10 years need to transition from parental control to self-control (5), which warrants substantial attention. Self-control for sufficient exercise time is necessary to maintain low serum lipid levels. We believe that focused intervention and education should be provided in preadolescence to foster self-control skills for securing exercise time.

##### 4.2.3.2 Rules for snacking

Proactively ensuring to not eat snacks while watching television may be associated with non-HDL-C levels below the median. Snacking while watching television undermines the intention to eat healthily (8). Moreover, consuming food while watching television and engaging in prolonged snacking leads to an increase in the quantity of food intake (31, 32). In Japan, eating snacks or meals while watching television is repeatedly emphasized as an “unhealthy behavior” from an early age. The absence of significant differences may be attributed to self-reporting bias, as children may perceive these behaviors as undesirable and respond accordingly. Adherence to snacking rules may contribute to maintaining low serum lipid levels. The study does not consider social and environmental factors such as snacks available at home or parental support to help children adhere to the rules for snacking, because it does not include the parents’ responses. Analyses incorporating this information will likely be necessary to further elucidate the relationship between adherence to the rules of children’s snacking and serum lipid levels. Although the association was borderline, we conclude that limiting snacking while watching television may be considered a feasible form of self-control strategy during the critical preadolescent period. Therefore, we conclude that the self-control of snacking among preadolescent children.

##### 4.2.3.3 Physical condition and mood on Mondays

Logistic regression analysis also showed a similar pattern as observed in the difference in non-HDL-C between Monday’s physical condition and mood, which was categorized as either very good or poor. While elevated serum lipid levels are commonly associated with an increased risk of disease, they may also contribute to alleviating discomfort on Mondays. Our hypothesis suggested that favorable conditions on Mondays are indicative of self-control of lifestyle behaviors over the weekend. We anticipated that a sense of wellbeing on Monday would correlate with lower serum lipid levels. However, the data did not support this hypothesis. Although a relationship between physical condition on Mondays and serum lipid levels was observed, this study was unable to conclusively determine that self-control of weekend lifestyle behaviors is a significant factor influencing preadolescent serum lipid levels. To low explanatory power (Nagelkerke R2) and potential unmeasured may have influenced this relationship.

##### 4.2.3.4 Weight and sex

Non-HDL-C levels below the median were associated with body weight below the median and with being male. This finding is consistent with previous studies showing that lower body weight and male sex are associated with lower serum lipid levels (14, 20). Supporting children in evaluating their serum lipid levels, given the biological characteristics of serum lipids is crucial. This support is essential for fostering the self-control of lifestyle behaviors to maintain low serum lipid values.

#### 4.2.4 Self-control of healthy lifestyle behaviors that should be focused on for preadolescence

In children aged 9–10 years, lifestyle behaviors associated with low serum lipid levels included ensuring sufficient exercise time; setting and adhering to rules for snacking may also be associated. These results support our hypothesis that self-control of lifestyle behaviors in preadolescent children contributes to maintaining low-serum lipid levels. Previous research has highlighted the significance of exercise among various lifestyle behaviors in enhancing health outcomes (28). Ensuring sufficient exercise time is beneficial for adults and children, because it aids in maintaining low serum lipid levels. The skill of controlling and ensuring time for exercise is instrumental in improving children’s current and future health outcomes. Furthermore, assisting children in setting and adhering to the rules for snacking is likely a crucial initial step in fostering self-control over their eating behaviors. Acquiring these self-control skills can contribute to the maintenance of low serum lipid levels in children and enhance their current and future health outcomes. These skills are crucial for fostering an independent and healthy lifestyle in the future and should be explicitly taught and reinforced during the preadolescent period.

This study is the first to identify specific daily lifestyle behaviors for children aged 9–10 years for maintaining low serum lipid levels that should be prioritized via self-control.

### 4.3 Limitations

First, this study relied on a self-administered questionnaire completed by children that has the possibility of self-reporting bias, because children may select exemplary answers of “healthy” behaviors as well as recall bias, as it may be difficult for children to recall and answer about behaviors from a few days ago (9). Measurement bias may occur due to the binary categorization of complex behaviors. This study used secondary data and did not include information about the parents that could influence children’s serum lipid levels, including parental responses regarding children’s lifestyle behaviors. The low effect size and explanatory power may be due to the inability to consider and adjust for confounding factors in the analysis. Second, as a cross-sectional study, a long-term longitudinal survey of the subjects in this study is needed to clarify the long-term effects of self-control of lifestyle behaviors on serum lipid levels. Third, the use of secondary data in this study, which were obtained through a municipal project, original questionnaires, and data, cannot be made public, thereby limiting the reproducibility of the research. The project targeted all fourth-grade children enrolled in public elementary schools within the municipality, to ensure equitable access to benefits and fairness in municipal services. Consequently, the findings of this study are reflective of the children in the targeted area. However, the results may not be directly applicable to urban areas or private elementary schools in other regions, because the project was conducted in public elementary schools located in depopulated regions. The findings can be further generalized by considering the characteristics of different regions and school types (public and private) and analyzing data from children in various regions or private schools.

### 4.4 Implications for clarity enhancement

This study is notable as it examined the self-control status of healthy lifestyle behaviors associated with serum lipid levels and identified specific behaviors that preadolescent children should focus on for their current and future health from among many lifestyle factors. In addition, at elementary schools or within communities, accurately gathering information such as information about their family, is often challenging due to privacy concerns. Therefore, health education or intervention typically focuses on a child’s response to the self-control status of lifestyle behaviors. Moreover, this study conducted a detailed analysis using data that can be collected on-site, and the findings of this research can be used in elementary schools or communities.

The findings of this study have substantial implications for public health and school health initiatives. In the realm of public health practice, continuing comprehensive educational efforts aimed at increasing residents’ awareness of the importance of having sufficient time for exercise and setting and adherence to the rules for snacking is imperative to maintain optimal serum lipid levels. Concurrently, cultivating environments that promote healthy behaviors, considering both “social and environmental influences, and implementing interventions and health education programs that enhance individuals’ skills in controlling and adapting healthy behaviors are essential. These initiatives are vital for advancing the overall health of the country’s population. Comprehensive enhancements in the social environment have been proposed to facilitate increased physical activity at the community level (4, 33). Moreover, the results underscore the importance of fostering self-control of lifestyle behavior among adults and children. Integrating knowledge and skills in elementary school health education can enhance children’s current and long-term health outcomes. Furthermore, interventions targeting social and environmental influences on behavior, such as raising parental awareness and organizing school events, can extend the impact of educational institutions to the wider community.

## 5 Conclusion

For preadolescent children, the lifestyle behaviors that should be managed to contribute to low serum lipid levels include ensuring and adjusting exercise time as well as setting and adhering to the rules for snacking. Supporting children to acquire the self-control skills of these lifestyle behaviors may maintain their serum lipid levels at low levels and improve their current and future outcomes.

## Data Availability

The data analyzed in this study is subject to the following licenses/restrictions: The data are not publicly available because of confidentiality reasons. Requests to access these datasets should be directed to Kotomi Yamashita, cotomiyama@hiroshima-u.ac.jp.

## References

[1] Truthmann J Schienkiewitz A Kneuer A Du Y Scheidt-Nave C. Tracking of serum lipids from prepuberty to young adulthood: results from the KiGGS cohort study. Lipids Health Dis. (2024) 23:421. doi: 10.1186/s12944-024-02409-1, PMID: 39725986 PMC11670486

[2] Gujral J Jyotsna G. Pediatric dyslipidemia. Treasure Island: StatPearls; (2025). Available online at: https://www.ncbi.nlm.nih.gov/books/NBK585106/?report=printable

[3] Mielke GI Brown WJ Wehrmeister FC Goncalves H Oliveira I Menezes AM . Associations between self-reported physical activity and screen time with cardiometabolic risk factors in adolescents: findings from the 1993 Pelotas (Brazil) birth cohort study. Prev Med Baltim. (2019) 119:31–6. doi: 10.1016/j.ypmed.2018.12.008, PMID: 30578907

[4] Glanz K Rimer BK Viswanath K. Health behavior: theory, research, and practice. 6th ed. Hoboken, NJ: Jossry-Bass A Wiley Brand; (2024).95–115 p.

[5] Baker S Morawska A Mitchell A. Promoting children’s healthy habits through self-regulation via parenting. Clin Child Fam Psychol Rev. (2019) 22:52–62. doi: 10.1007/s10567-019-00280-6, PMID: 30725307

[6] Miller AL Gearhardt AN Fredericks EM Katz B Shapiro LF Holden K . Targeting self-regulation to promote health behaviors in children. Behav Res Ther. (2018) 101:71–81. doi: 10.1016/j.brat.2017.09.008, PMID: 29050636 PMC5801044

[7] Schulz van Endert T. Addictive use of digital devices in young children: associations with delay discounting, self-control and academic performance. PLoS One. (2021) 16:e0253058. doi: 10.1371/journal.pone.0253058, PMID: 34157026 PMC8219150

[8] Dohle S Diel K Hofmann W. Executive functions and the self-regulation of eating behavior: a review. Appetite. (2018) 124:4–9. doi: 10.1016/j.appet.2017.05.041, PMID: 28551113

[9] Mann T Ward A. The self-control of eating. Annu Rev Psychol. (2025) 76:87–114. doi: 10.1146/annurev-psych-012424-39094058

[10] Bundy DAP de Silva N Horton S Patton GC Schultz L Jamison DT . Investment in child and adolescent health and development: key messages from disease control priorities, 3rd edition. Lancet. (2018) 391:687–99. doi: 10.1016/S0140-6736(17)32417-0 29153316

[11] Tremblay MS Carson V Chaput JP Connor Gorber S Dinh T Duggan M . Canadian 24-hour movement guidelines for children and youth: an integration of physical activity, sedentary behaviour, and sleep. Appl Physiol Nutr Metab. (2016) 41:S311–27. doi: 10.1139/apnm-2016-0151 27306437

[12] World Health Organization Who guidelines on physical activity and sedentary behaviour (2020). Geneva: WHO publications. 33369898

[13] Austin PC Steyerberg EW. Events per variable (EPV) and the relative performance of different strategies for estimating the out-of-sample validity of logistic regression models. Stat Methods Med Res. (2017) 26:796–808. doi: 10.1177/0962280214558972, PMID: 25411322 PMC5394463

[14] Dobashi K. Changes in serum cholesterol in childhood and its tracking to adulthood. J Atheroscler Thromb. (2022) 29:5–7. doi: 10.5551/jat.ED163, PMID: 33612708 PMC8737080

[15] Eissa MA Mihalopoulos NL Holubkov R Dai S Labarthe DR. Changes in fasting lipids during puberty. J Pediatr. (2016) 170:199–205. doi: 10.1016/j.jpeds.2015.11.018, PMID: 26706233 PMC4769904

[16] Kawasaki H Yamasaki S Shintaku H Fukita S. Identification of factors influencing cholesterol changes in elementary-school children: a longitudinal study. Children. (2022) 9:518. doi: 10.3390/children9040518, PMID: 35455562 PMC9026368

[17] Guo LL Chen YQ Lin QZ Tian F Xiang QY Zhu LY . Non-HDL-C is more stable than LDL-C in assessing the percent attainment of non-fasting lipid for coronary heart disease patients. Front Cardiovasc Med. (2021) 8:8. doi: 10.3389/fcvm.2021.649181, PMID: 33869310 PMC8049565

[18] Saquib J Saquib N Chamsi Basha A Aljundi S Rajab AM Rajab TM . The associations of family atmosphere, religiosity and lifestyle with self-esteem and self-control among Saudi adolescents. Int J Psychol. (2024) 59:1245–53. doi: 10.1002/ijop.13250, PMID: 39340172

[19] Verbeek R Hovingh GK Boekholdt SM. Non-high-density lipoprotein cholesterol: current status as cardiovascular marker. Curr Opin Lipidol. (2015) 26:502–10. doi: 10.1097/MOL.0000000000000237, PMID: 26780004

[20] Bouhour S Plantefève R Gillet V Abolghasemi A Bouchouirab FZ Baccarelli AA . Establishing non-fasting reference values for plasma lipids levels based on age, sex, and puberty stage in a French-Canadian pediatric population. Lipids Health Dis. (2024) 23:54. doi: 10.1186/s12944-024-02040-0, PMID: 38388929 PMC10882849

[21] Katz MH. Multivariable analysis: a practical guide for clinicians and public health researchers. New York: Cambridge University Press (2011).

[22] Rollo S Antsygina O Tremblay MS. The whole day matters: understanding 24-hour movement guideline adherence and relationships with health indicators across the lifespan. J Sport Health Sci. (2020) 9:493–510. doi: 10.1016/j.jshs.2020.07.004, PMID: 32711156 PMC7749249

[23] Gadiraju TV Patel Y Gaziano JM Djoussé L. Fried food consumption and cardiovascular health: a review of current evidence. Nutrients. (2015) 7:8424–30. doi: 10.3390/nu7105404 26457715 PMC4632424

[24] Dangal A Tahergorabi R Acharya D Timsina P Rai K Dahal S . Review on deep-fat fried foods: physical and chemical attributes, and consequences of high consumption. Eur Food Res Technol. (2024) 250:1537–50. doi: 10.1007/s00217-024-04482-3

[25] Grootveld M. Evidence-based challenges to the continued recommendation and use of peroxidatively-susceptible polyunsaturated fatty acid-rich culinary oils for high-temperature frying practises: experimental revelations focused on toxic aldehydic lipid oxidation products. Front Nutr. (2022) 8:711640. doi: 10.3389/fnut.2021.711640 35071288 PMC8769064

[26] Lee W Kang C Park C Bell ML Armstrong B Roye D . Association of holidays and the day of the week with suicide risk: multicountry, two stage, time series study. BMJ. (2024) 387:e077262. doi: 10.1136/bmj-2024-077262, PMID: 39442941 PMC11497772

[27] Jones PR Rajalahti T Resaland GK Aadland E Steene-Johannessen J Anderssen SA . Associations of physical activity and sedentary time with lipoprotein subclasses in Norwegian schoolchildren: the active smarter kids (ASK) study. Atherosclerosis. (2019) 288:186–93. doi: 10.1016/j.atherosclerosis.2019.05.023, PMID: 31200940

[28] Wilhite K Booker B Huang BH Antczak D Corbett L Parker P . Combinations of physical activity, sedentary behavior, and sleep duration and their associations with physical, psychological, and educational outcomes in children and adolescents: a systematic review. Am J Epidemiol. (2023) 192:665–79. doi: 10.1093/aje/kwac212, PMID: 36516992 PMC10089066

[29] Zhu X Healy S Haegele JA Patterson F. Twenty-four-hour movement guidelines and body weight in youth. J Pediatr. (2020) 218:204–9. doi: 10.1016/j.jpeds.2019.11.031, PMID: 31959469 PMC7042069

[30] del Pozo-Cruz J García-Hermoso A Alfonso-Rosa RM Alvarez-Barbosa F Owen N Chastin S . Replacing sedentary time: meta-analysis of objective-assessment studies. Am J Prev Med. (2018) 55:395–402. doi: 10.1016/j.amepre.2018.04.042, PMID: 30122216

[31] Garg D Smith E Attuquayefio T. Watching television while eating increases food intake: a systematic review and meta-analysis of experimental studies. Nutrients. (2025) 17:116. doi: 10.3390/nu17010166, PMID: 39796600 PMC11722569

[32] Tripicchio GL Bailey RL Davey A Croce CM Fisher JO. Snack frequency, size, and energy density are associated with diet quality among US adolescents. Public Health Nutr. (2023) 26:2374–82. doi: 10.1017/S1368980023001635, PMID: 37548183 PMC10641603

[33] Padmapriya N Bernard JY Tan SYX Chu AHY Goh CMJL Tan SL . The prospective associations of 24-hour movement behaviors and domain-specific activities with executive function and academic achievement among school-aged children in Singapore. Front Public Health. (2024) 12::1412634. doi: 10.3389/fpubh.2024.1412634, PMID: 39296832 PMC11409845

